# Development and validation of a multiplex UHPLC‐MS/MS assay with stable isotopic internal standards for the monitoring of the plasma concentrations of the antiretroviral drugs bictegravir, cabotegravir, doravirine, and rilpivirine in people living with HIV

**DOI:** 10.1002/jms.4506

**Published:** 2020-03-11

**Authors:** Perrine Courlet, Susana Alves Saldanha, Matthias Cavassini, Catia Marzolini, Eva Choong, Chantal Csajka, Huldrych F. Günthard, Pascal André, Thierry Buclin, Vincent Desfontaine, Laurent Arthur Decosterd

**Affiliations:** ^1^ Service of Clinical Pharmacology Lausanne University Hospital and University of Lausanne Lausanne Switzerland; ^2^ Service of Infectious Diseases Lausanne University Hospital and University of Lausanne Lausanne Switzerland; ^3^ Division of Infectious Diseases and Hospital Epidemiology University Hospital of Basel Basel Switzerland; ^4^ University of Basel Basel Switzerland; ^5^ Centre for Research and Innovation in Clinical Pharmaceutical Sciences University Hospital and University of Lausanne Lausanne Switzerland; ^6^ Institute of Pharmaceutical Sciences of Western Switzerland University of Geneva, University of Lausanne Geneva Switzerland; ^7^ School of Pharmaceutical Sciences University of Geneva Geneva Switzerland; ^8^ Division of Infectious Diseases and Hospital Epidemiology University Hospital Zurich Switzerland; ^9^ Institute of Medical Virology, Swiss National Reference Centre for Retroviruses University of Zurich Zurich Switzerland

**Keywords:** antiretroviral therapy, long‐acting injectables, pharmacokinetics, therapeutic drug monitoring, UHPLC‐MS/MS

## Abstract

The widespread use of highly active antiretroviral treatments has dramatically changed the prognosis of people living with HIV (PLWH). However, such treatments have to be taken lifelong raising issues regarding the maintenance of both therapeutic effectiveness and long‐term tolerability. Recently approved or investigational antiretroviral drugs present considerable advantages, allowing once daily oral dosage along with activity against resistant variants (eg, bictegravir and doravirine) and also parenteral intramuscular administration that facilitates treatment adherence (eg, long‐acting injectable formulations such as cabotegravir and rilpivirine). Still, there remains a risk of insufficient or exaggerated circulating exposure due to absorption issues, abnormal elimination, drug‐drug interactions, and others. In this context, a multiplex ultra‐high performance liquid chromatography coupled to tandem mass spectrometry (UHPLC‐MS/MS) bioassay has been developed for the monitoring of plasma levels of bictegravir, cabotegravir, doravirine, and rilpivirine in PLWH. A simple and convenient protein precipitation was performed followed by direct injection of the supernatant into the UHPLC‐MS/MS system. The four analytes were eluted in less than 3 minutes using a reversed‐phase chromatography method coupled with triple quadrupole mass spectrometry detection. This bioassay was fully validated following international guidelines and achieved good performances in terms of trueness (94.7%‐107.5%), repeatability (2.6%‐11%), and intermediate precision (3.0%‐11.2%) over the clinically relevant concentration ranges (from 30 to 9000 ng/mL for bictegravir, cabotegravir, and doravirine and from 10 to 1800 ng/mL for rilpivirine). This sensitive, accurate, and rapid UHPLC‐MS/MS assay is currently applied in our laboratory for routine therapeutic drug monitoring of the oral drugs bictegravir and doravirine and is also intended to be applied for the monitoring of cabotegravir/rilpivirine levels in plasma from PLWH receiving once monthly or every 2‐month intramuscular injection of these long‐acting antiretroviral drugs.

## 1 INTRODUCTION

Optimal efficacy and good tolerability are key points during the development of antiretroviral (ARV) drugs.^1^ Yet, besides therapeutic effectiveness and drug safety profile, long‐term adherence is required to achieve viral suppression.^2^, ^3^ The development of long‐acting injectable (LAI) formulations can overcome the adherence issue^4^ by maintaining effective plasma concentrations over months. Thus, LAI has the potential to improve adherence thereby preventing drug resistance. In addition, LAI can improve patients' privacy and reduce social stigmas associated with daily intake of ARV drugs. It has been stated that about as much as 50% to 70% of people living with HIV (PLWH) would be interested in LAI formulations when available.^5^

Cabotegravir and rilpivirine are the first two drugs of LAI formulation, currently in final phase of clinical development.^6^, ^7^ Cabotegravir is a potent HIV integrase strand transfer inhibitor (INSTI),^8^ while rilpivirine is non‐nucleoside HIV reverse transcriptase inhibitor (NNRTI). Long plasma half‐life of both substances made them good candidates for the development of LAI formulations administered monthly^9^, ^10^ or every 2 months.^11^ In addition to HIV treatment, LAI‐ARV drugs are also investigated separately in the indication of pre‐exposure prophylaxis (PreP). Whether used for treatment or prevention, important pharmacokinetic variability was shown following intramuscular injection of cabotegravir and rilpivirine in clinical trials.^9^, ^10^, ^12^, ^13^, ^14^ These clinical studies have generally included carefully selected PLWH, who may not reflect the complex situation in a real‐life clinical setting. In particular, drug‐drug interactions (DDIs) are likely to occur,^15^ also with LAI‐ARV drugs, and we have at present very limited information on their actual clinical importance, prompting the monitoring of ARV plasma levels when new comedications at risk of DDIs are introduced in patients on LAI‐ARV drugs. Besides, intersubject variability may be more pronounced particularly in special population (ie, underweight or obese patients, hepatic or renal impairment, aging, or pregnancy).

In addition to these novel injectable formulations, ARV developments are also focused on improving the safety and tolerability profile. The last‐generation ARV drugs bictegravir (a potent unboosted INSTI^16^) and doravirine (a next‐generation NNRTI^17^) represent attractive oral therapeutic options because of their improved tolerability profiles. Both bictegravir and doravirine are substrates of CYP3A4 and can consequently be victims of DDIs. However, there is currently a lack of data concerning the magnitude of DDIs with these novel ARV drugs. Yet, in the next few years, most PLWH in middle‐ and high‐income countries will switch to one of these last‐generation ARV therapies, either oral or LAI formulations.

The availability of liquid chromatography coupled to tandem mass spectrometry (LC‐MS/MS) methodologies for the determination of ARV concentrations in human plasma is a key aspect for drug pharmacokinetic studies and therapeutic drug monitoring (TDM) in patients. Several assays have been previously developed for the measurement of rilpivirine as oral formulation.^18^, ^19^, ^20^ To the best of our knowledge, only two LC‐MS/MS assays have been published for the quantification of bictegravir in human plasma.^21^, ^22^ In addition, although cabotegravir and doravirine plasma concentrations have been determined in several studies,^23^, ^24^ no publication has been dedicated to the development and the validation of such LC‐MS/MS methodologies.

In this article, we aimed at developing and validating a simple and fast multiplex assay by ultrahigh‐performance liquid chromatography coupled to tandem mass spectrometry (UHPLC‐MS/MS) for the simultaneous determination of the latest generation ARV drugs bictegravir, cabotegravir, doravirine, and rilpivirine in human plasma.

## 2 MATERIAL AND METHODS

### 2.1 Chemical and reagents

**Figure 1:**
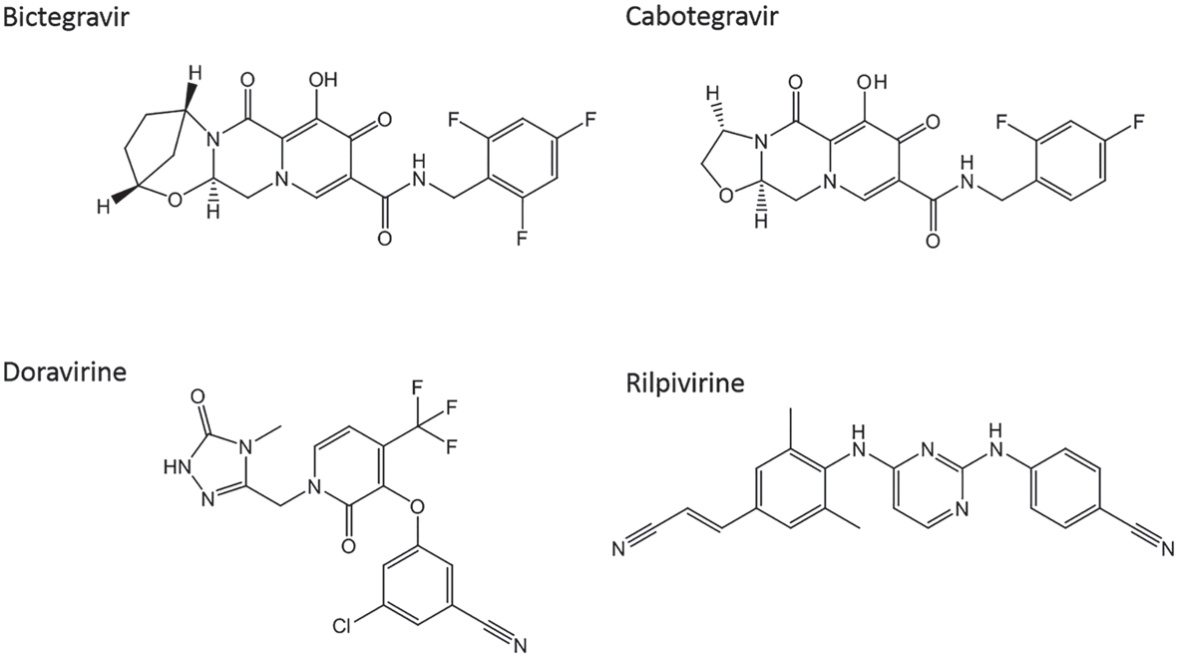
Chemical structures of the analyzed antiretroviral drugs

Bictegravir (purity ≥98%) and cabotegravir (≥98%) were obtained from Alsachim (Strasbourg, France), while doravirine (98%) and rilpivirine (98.5%) were purchased from Toronto Research Chemical (Toronto, Canada). Chemical structures are depicted in Figure 1. Their stable isotopically labelled internal standards (ISs) (ie, [^13^C_,_^2^H_2,_^15^N]‐bictegravir [purity ≥98%], [^13^C_,_^2^H_5_]‐cabotegravir [≥95%], [^13^C_6_]‐doravirine [≥95%], and [^13^C_6_]‐rilpivirine [99.3%]), were obtained from Alsachim. In addition, cabotegravir O‐β‐d‐glucuronide (purity 95%) was purchased from Alsachim.

All solvents (ie, acetonitrile [ACN], methanol [MeOH], and formic acid [FA] [98%‐100%]) were of analytical grade and were obtained from Merck (Darmstadt, Germany). Dimethylsulfoxide (DMSO, 99.5%) was purchased from Alfa Aesar (Kandel, Germany). Ultrapure water was supplied by a Milli‐Q UF‐Plus apparatus (Millipore Corp, Burlington, MA, USA).

Human blank plasma samples used for method development and validation, as well as for the preparation of calibration samples and quality controls (QCs), were obtained according to institutional ethical standard from citrated blood from patients with *polycythemia vera* who underwent regular phlebotomy at the Center of Transfusion Medicine, Lausanne University Hospital, Lausanne, Switzerland, by centrifugation (1970*g*, ie, 3000 rpm, 10 min, +4°C, Hettich model Rotanta 460RF centrifuge) or from TCS Bioscience (Buckingham, UK).

### 2.2 Stock solutions preparation

Each analyte was weighed and dissolved in the required volume of solvent. Stock solutions of bictegravir (1 or 5 mg/mL), cabotegravir (1 mg/mL), and doravirine (2 mg/mL) were prepared in DMSO. Rilpivirine powder was dissolved in a mixture of DMSO:MeOH 1:1 to obtain the final concentration of 0.5 mg/mL. These stock solutions were stored at −20°C for bictegravir, cabotegravir, and doravirine. The stock solution of rilpivirine was stored at +4°C, as currently done for the routine monitoring of rilpivirine plasma concentrations in the framework of our TDM service.^18^

One working solution (WS) at 100 μg/mL for bictegravir, cabotegravir, and doravirine and 20 μg/mL for rilpivirine was prepared in a mixture of H_2_O:DMSO (3:1) for calibration standards. Another WS for validation standards was independently prepared in the same solvent, at the following concentrations: 90 μg/mL for all analytes except for rilpivirine (18 μg/mL). Spiking solutions for calibration and validation samples were prepared at the appropriate concentrations by sequentially diluting the WSs in a mixture of H_2_O:DMSO (3:1).

Stock solutions of each IS were prepared at 1 mg/mL in DMSO (isotopically labelled bictegravir and cabotegravir) or MeOH (isotopically labelled rilpivirine and doravirine) and stored at −20°C. An IS‐WS was prepared at 250 ng/mL for all analytes, except for rilpivirine (50 ng/mL) by mixing the required volumes of the four IS stock solutions with a mixture of MeOH:ACN 1:1.

All solutions were stored at −20°C.

### 2.3 Calibration and validation standards preparation

Spiked plasma was obtained by diluting tenfold the spiking solutions (100 μL) with blank plasma (900 μL). The total added volume was ≤10% of the biological sample volume to follow the recommendations for bioanalytical method validation.^25^, ^26^ Nine concentration levels (k) of calibration samples were prepared each validation day (n = 3) at the following concentrations: 10 000, 5000, 2500, 1000, 500, 250, 125, 50, and 25 ng/mL, except for rilpivirine with fivefold lower concentrations (ie, 2000, 1000, 500, 200, 100, 50, 25, 10, and 5 ng/mL), with respect to the values commonly observed in clinical practice.^14^, ^27^, ^28^ Eight validation standards were prepared at the following concentrations: 9000, 4500, 1500, 600, 300, 150, 60, and 30 ng/mL, except for rilpivirine, having fivefold lower values. For each analyte, accurate determination of lower limits of quantifications (LLOQs) relied on the use of one validation sample at the estimated LLOQ and one at twofold to threefold LLOQ.

### 2.4 Plasma pre‐treatment procedure

Protein precipitation was operated by adding a 300‐μL volume of the IS‐WS to 100 μL of calibration, validation, or patient plasma samples. The mixture was vortexed and centrifuged at 18 620*g* (14 000 rpm) at +4°C for 10 minutes with a benchtop centrifuge (Benchtop Mikro 220R centrifuge, Hettich, Bäch, Switzerland). Three hundred microliters of the supernatant were directly transferred into an HPLC vial with insert.

### 2.5 UHPLC‐MS/MS instrumentation

UHPLC‐MS/MS analyses were conducted using an Ultimate 3000 Rapid Separation (RS) LC system (Thermo Fisher Scientific, San Jose, CA, USA) composed of an Ultimate 3000 RS column compartment, an RS autosampler, and an RS binary pump. Chromatographic separation was carried out with a Xselect HSS T3 analytical column from Waters (Milford, MA, USA) with 3.5‐μm particle size and dimensions of 2.1 × 75 mm. The UHPLC system was coupled with a TSQ Quantis triple quadrupole mass spectrometer from Thermo Fisher Scientific, equipped with an OptaMax NG ion source used in electrospray ionization (H‐ESI) mode. Data acquisition, treatment, and instrument control were performed using the XCalibur software version 4.1.31.9 and Chromeleon version DCMS link (ThermoFisher Scientific).

### 2.6 Analytic conditions

The mobile phases (ie, H_2_O + 0.1% FA (A) and ACN + 0.1% FA (B)) were delivered at a flow rate of 300 μL/min, following this multistep gradient: first, linear gradient from 40% to 60% B in 3 minutes, up to 95% B in 0.2 minutes, followed by an isocratic stage at 95% B for 0.8 minutes. Then, solvent B was reduced to 40% (initial conditions) in 0.1 minute, followed by a re‐equilibration step up to 5 minutes (total analysis time). Samples were stored at +5°C in the autosampler, and the injection volume was 7 μL.

Polarity switching capability enabled ESI positive (spray voltage 3900 V) and negative (spray voltage 3400 V) analysis in the same sample injection. ESI source parameters were optimized as follows: the ion transfer tube and vaporizer temperatures at 300°C and 150°C, respectively; sheath, auxiliary, and sweep gas flow rates at 45, 25, and 0 (arbitrary units), respectively. The first (Q1) and third (Q3) quadrupoles operated with a mass resolution of 1.2 Da (ie, *m/z* 1.2 full width at half maximum, FWHM). The cycle time was 0.2 seconds. The pressure of the collision gas (argon) in the second quadrupole (Q2) was set at 1.5 mTorr.

### 2.7 Validation procedure

#### 2.7.1 Selectivity

The selectivity of the method was evaluated by analyzing blank human plasma from 10 different donors processed with pure ACN:MeOH 1:1. Cross‐talk interferences were then established by injecting a high concentration calibration sample processed with pure ACN:MeOH 1:1 (no ISs) and a blank plasma processed with the IS‐WS. Finally, the injection of blank solvent (ACN:MeOH 1:1) or blank plasma extract immediately after a high calibration sample processed with IS‐WS allowed the assessment of carryover effect.

In addition, since the INSTIs bictegravir and cabotegravir are metabolized by UDP‐glucuronosyltransferase (UGT) to glucurono‐conjugated metabolites, the separations between drug and their metabolites were investigated. This was particularly important to ascertain that these INSTIs and their respective glucuronides do not coelute that would give spuriously high drug levels because of the in‐source dissociation of glucuronide to parent compound during the ionization step, such as previously reported for raltegravir.^29^, ^30^ Cabotegravir glucuronide was provided by Alsachim, whereas bictegravir glucuronide was not available at the time of the current development. MS/MS transition of cabotegravir glucuronide was assessed by direct infusion into the MS detector. Bictegravir glucuronide MS/MS transition was empirically reckoned, considering that bictegravir was the main fragment obtained from bictegravir glucuronide. The selectivity of the method regarding glucuronides was evaluating by injecting a plasma sample containing cabotegravir and cabotegravir glucuronide processed with blank MeOH:ACN (1:1) and a plasma sample from an HIV‐infected patient receiving bictegravir.

#### 2.7.2 Matrix effect, extraction recovery, and process efficiency

##### Qualitative evaluation of matrix effect

The method proposed by Bonfiglio et al^31^ allowed the evaluation of the potential impact of endogenous compounds on ionization process. A solution of analytes in MeOH (1100 ng/mL for bictegravir, cabotegravir, and doravirine and 200 ng/mL for rilpivirine) and ISs (200 ng/mL for all IS except for [^13^C_6_]‐rilpivirine at 50 ng/mL) was continuously infused postcolumn, while seven different blank plasma extracts processed with pure ACN:MeOH 1:1 were injected into the UHPLC‐MS/MS system. Each MS/MS transition was visually examined to check for any alteration (suppression or enhancement) at the analytes' retention times.

##### Quantitative assessment of matrix effect, extraction recovery, and process efficiency

Matrix effects (MEs), extraction recoveries (ERs), and process efficiencies (PEs) were quantitatively evaluated following Matuszeswski's approach.^32^ Low (60 ng/mL for bictegravir, cabotegravir, and doravirine and 12 ng/mL for rilpivirine), medium (600 ng/mL for bictegravir, cabotegravir, and doravirine and 120 ng/mL for rilpivirine), and high (6000 ng/mL for bictegravir, cabotegravir, and doravirine and 1200 ng/mL for rilpivirine) concentrations were considered. Three sets of samples at the three concentration levels were prepared as follows: (A) three neat solutions (H_2_O) with analytes and ISs; (B) seven postextraction spiked blank plasma in duplicate; (C) seven pre‐extraction spiked blank plasma in duplicate. IS normalization was considered by using ratio of analyte peak areas to the corresponding IS peak area to calculate the following parameters: IS‐normalized matrix effects (IS‐nMEs) as B/A (in %), IS‐normalized extraction recoveries (IS‐nERs) as C/B (in %), and IS‐normalized process efficiencies (IS‐nPEs) as C/A (in %).

Relative standard deviation (RSD) of slopes from linear regressions estimated at L, M, and H concentrations were also calculated. An LC‐MS/MS method is considered devoid of significant ME if RSD value is <4%.

#### 2.7.3 Trueness, precision, accuracy profiles, limits of quantification, and linearity

Trueness and precision of the method were assessed over three different days. Several regression models were fitted to adequately describe the response concentration profile. The selection of the best calibration model was based on the estimations of trueness and precision, the narrowest β‐expectation tolerance interval, and the lowest LLOQ.^33^

Concentrations of the validation standards were back‐calculated with the daily calibration curve. The trueness (systematic error) was defined as the percentage of deviation between the calculated concentrations of validation standards and the nominal value. The precision (random error) was estimated by two components: the repeatability (intraday variances) and intermediate precision (intraday and interday variances).^34^, ^35^, ^36^ Precision parameters were reported as RSD at each concentration level.^33^ The total error encompassed both systematic and random errors and was evaluated using accuracy profiles. β‐expectation tolerance intervals represent the concentration range where β% of future results is expected to lie.^37^, ^38^, ^39^ Using data obtained during the validation phase, this approach allows to confidently predict the future results that will be obtained during the routine use of the method. Based on the absolute accuracy profiles, LLOQ was graphically interpolated as the lowest concentration for which the β‐expectation tolerance interval crosses the acceptance limits (±30%).^25^, ^26^, ^40^

Finally, the capacity of the method to give quantitative results proportional to nominal concentrations was evaluated by ordinary least square regression on the plot representing back‐calculated concentrations vs nominal concentrations. This defines the linearity of trueness and was assessed each day of validation.

#### 2.7.4 Measurement uncertainty

An analytical result should also be reported with respect to its measurement uncertainty (MU). MU was evaluated by the type A estimation method, based on experimental measurements. Feinberg et al demonstrated that the β*‐*expectation tolerance interval is directly related to the MU.^41^ The accuracy profile validation methodology enables the estimation of MU without any additional experiments.^42^ MU can be derived from the data collected during the validation phase, by fixing the *β* value at 0.95. Continuous models were developed in order to obtain values of MU as a function of the concentration of the analytes. Several models were tested to identify the one that fitted the data best, by visual inspection of the uncertainty profiles. Ordinary least squares regression was used to estimate the coefficients of the uncertainty function. This methodology allows easy calculation of the MU at any concentration within the validation domain. All calculations were performed using Excel.

#### 2.7.5 Stability studies

Stability studies included bench‐ and long‐term stabilities. The stability of plasma at room temperature (RT) and in the fridge (+4°C) up to 48 hours was evaluated. In addition, stability after three freeze/thaw cycles was assessed by thawing frozen samples at RT for 1 hour and refreezing them during 1 hour, three times in a row. Furthermore, plasma samples were submitted to thermal viro‐inactivation process (60 min at +60°C in a water bath) since this procedure has been shown to efficiently inactivate HIV particles present in the samples.^43^, ^44^ Finally, medium stability was evaluated with plasma samples frozen at −20°C and −80°C during 6 weeks. Analyses were performed in triplicate. The mean of the concentrations obtained after each stability study were compared with the mean concentration of samples prepared at time 0.

### 2.8 Patients samples

Blood samples were collected from PLWH at the request of physicians during their usual follow‐up visits. In the frame of the hospital routine TDM program for ARV drugs, TDM was performed rather liberally, being particularly recommended in case of suspicion of altered pharmacokinetics (eg, DDIs or impaired hepatic/renal functions) or to evaluate short‐term adherence to oral ARV drugs. Blood samples were collected in EDTA‐Monovettes. The preanalytical sample preparation was performed in our laboratory by centrifuging the Monovettes, transferring plasma into propylene tubes in class II biohazard hoods using standard biosafety precautions (gloves and others) and storing samples at −20°C until batch analyses.

## 3 RESULTS

### 3.1 Analytical method development

The optimization of the LC‐MS/MS assay aimed at improving sensitivity while minimizing runtime. First, standard solutions of each analyte at 5 μg/mL in MeOH were directly infused into the MS detector in order to select optimal MS/MS, as reported in Table 1. LC‐MS/MS transitions for bictegravir and rilpivirine differed from reported values. In the only published bioassays for bictegravir, mass spectrometer operated in ESI positive mode, while in our study, bictegravir sensitivity was higher in the negative mode.^21^, ^22^ In most of published methods for the quantification of rilpivirine, MS/MS transition was 367/195 in the positive mode.^18^, ^20^, ^45^ However, the infusion of a rilpivirine solution into the MS detector revealed a higher sensitivity in the negative mode. This was certainly due to the fact that one main fragment with high intensity was observed in ESI− whereas multiple fragments with shared intensities were present after fragmentation in ESI+. Nevertheless, transition 367/195 in the positive mode was tested during method development, and the lower sensitivity was confirmed. Therefore, transition 365/142 in the negative mode was finally retained for the quantification of rilpivirine in this bioanalytical assay. Since no LC‐MS/MS method had been yet reported for the determination of cabotegravir and doravirine, no comparison between transitions could be made.

**Table 1:** MS/MS parameters and typical retention times of the four ARV drugs and their respective stable isotope–labelled ISs

Compound	ESI polarity (+/−)	Precursor Ion (*m/z*)	Product Ion (*m/z*)	Collision Energy (V)	Typical Retention Time, min
Bictegravir	−	448.4	286.2	25	2.16
	−		301.0	25	
[^13^C,^2^H_2_,^15^N]‐Bictegravir	−	452.2	203.0	35	2.13
	−		287.1	25	
Cabotegravir	−	404.2	305.1	26	1.74
	−		374.1	22	
[^13^C,^2^H_5_]‐Cabotegravir	−	410.2	311.1	27	1.72
	−		380.0	23	
Doravirine	+	426.1	111.9	24	2.35
	+		315.1	18	
[^13^C_6_]‐Doravirine	+	432.5	320.8	10	2.35
	+		416.4	11	
Rilpivirine	−	365.1	142.1	30	1.41
[^13^C_6_]‐Rilpivirine	−	371.2	148.1	31	1.41
	−		329.3	25	

Abbreviations: ARV, antiretroviral; IS, internal standard; MS/MS, tandem mass spectrometry.

**Figure 2:**
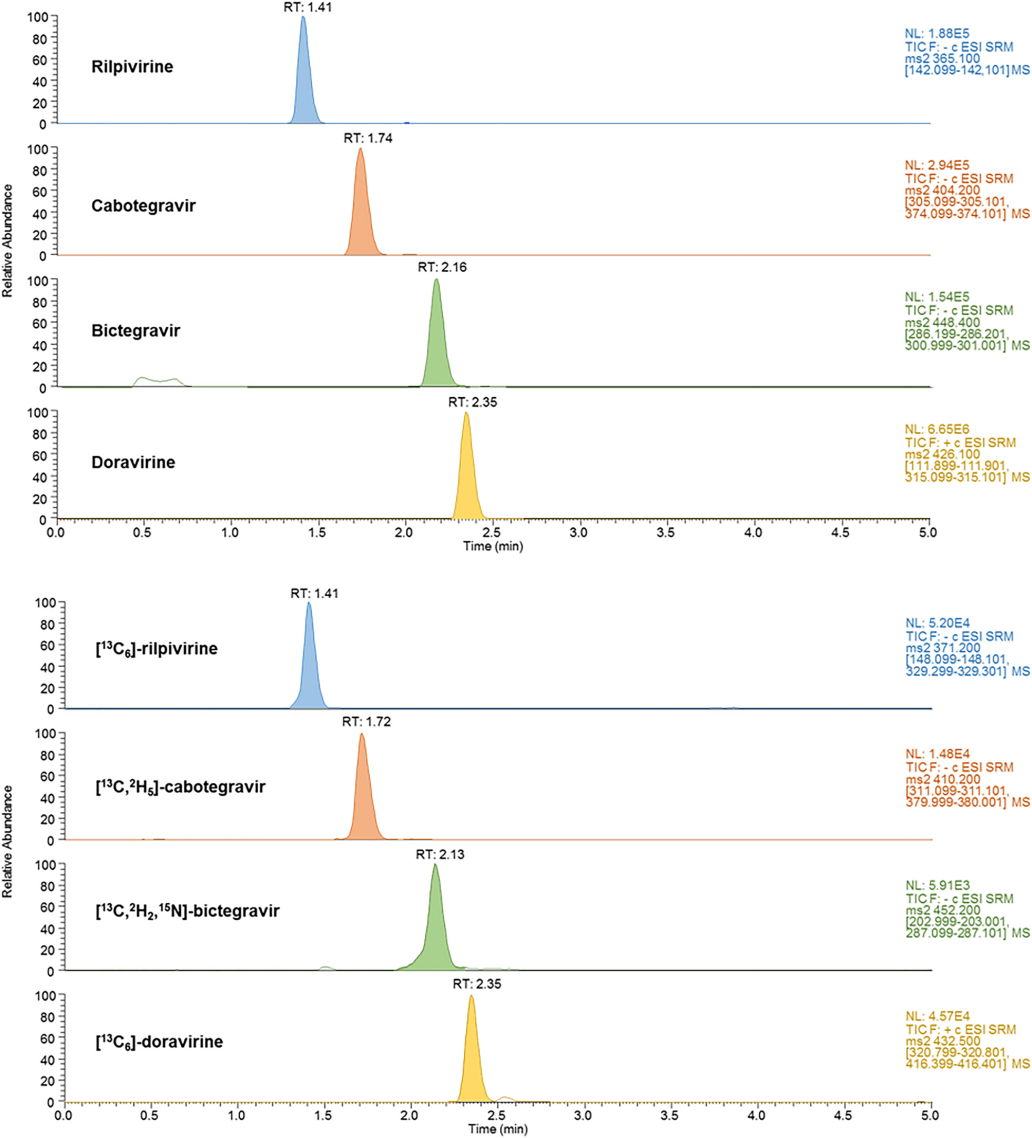
Ultrahigh‐performance liquid chromatography coupled to tandem mass spectrometry (UHPLC‐MS/MS) separation of a calibration sample containing the four antiretroviral drugs, at the concentration of 10 000 ng/mL for all the analytes except for rilpivirine (2000 ng/mL). Calibration sample was prepared as described in Section 2 [Colour figure can be viewed at wileyonlinelibrary.com]

Concerning the chromatographic part of the method, analytical efforts have been made to achieve satisfactory separation and peak shape, in order to accurately quantify each analyte. For that purpose, conventional mobile (ie, H_2_O + 0.1% FA and ACN + 0.1% FA) and stationary (Xselect HSS T3 column) phases were shown to be suitable. Mobile‐phase gradient program was optimized to adequately separate each analyte in a minimal runtime. Sample preparation was limited to a convenient and fast protein precipitation, which was considered sufficient to accurately quantify the range of concentrations commonly observed in clinical practice. Sensitivity was compared between different protein precipitation solvents, and a mixture of MeOH:ACN 1:1 was selected instead of MeOH or ACN. Different injection volumes of pretreated samples ranging from 3 to 10 μL were tested, and a volume of 7 μL was finally chosen as the best compromise between suitable sensitivity and satisfactory peak shape. As shown in Figure 2, an adequate separation of the four analytes was achieved in less than 3 minutes, with satisfactory peak shapes.

The MS part of the analytical assay was optimized (as reported in Section 2.6) by choosing the appropriate ESI source parameters to improve sensitivity while minimizing background noise. Finally, IS concentrations were selected to obtain satisfying IS‐normalized response functions, by avoiding variability due to low IS concentrations and by circumventing a significant contribution of IS signal to analyte signal in case of excessive IS concentrations.

### 3.2 Validation of the method

#### 3.2.1 Selectivity and carryover

The good selectivity of the chromatographic method was demonstrated with the absence of interference at the retention times of the four analytes when analyzing human blank plasma from 10 different sources.

The injections of a blank plasma processed with IS‐WS or the highest calibration standard processed with MeOH:ACN (1:1) did not reveal any significant signal on the analytes or IS transitions, respectively, demonstrating the absence of cross talks.

The additional experiments regarding the selectivity with the glucurono‐conjugated metabolites demonstrated that retention times for cabotegravir glucuronide and bictegravir glucuronide were 0.83 and 0.89 minutes, respectively. In consequence, they were adequately separated from their respective parent drug, cabotegravir and bictegravir, eluting at 1.74 and 2.16 minutes, respectively.

Carryover was considered satisfactory since the chromatogram of blank matrix samples or MeOH directly injected after the highest calibration standard was devoid of analytes' and IS traces.

#### 3.2.2 ME, ER, and PE

**Figure 3:**
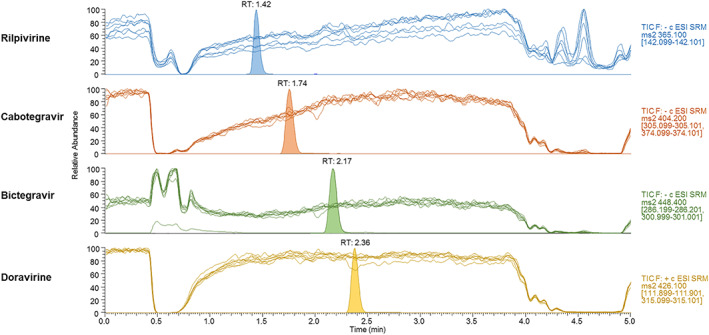
Qualitative assessment of matrix effect. Overlaid ultrahigh‐performance liquid chromatography coupled to tandem mass spectrometry (UHPLC‐MS/MS) profiles obtained from seven blank plasma extracts during postcolumn infusion of a solution containing the four analytes, as described in Section 2.7.2. Chromatographic peaks obtained during experiments were superimposed for interpretation [Colour figure can be viewed at wileyonlinelibrary.com]

As shown in Figure 3, no major interferences (ie, ion suppression or enhancement) were observed at analytes' retention times. This result supports the suitability of the chromatographic method, preventing an impact of endogenous plasma components on the ionization process of the four analytes and ISs.

Quantitative results of the assessment of IS‐nME, IS‐nER, and is‐nPE are summarized in Table 2. The IS‐nME of the analytes was considered satisfactory and varied from −6% to 12%, while RSD values were lower than 6%. Regarding IS‐nER and IS‐nPE, acceptable results were observed with values ranging from −15% to 4% and −16% to 5%, respectively, with RSD lower than 10%. Overall, matrix‐matched calibration along with the use of isotopically labelled IS was found to adequately limit MEs issues.

**Table 2:** Internal standard–normalized matrix effect (IS‐nME), extraction recovery (IS‐nER), and process efficiency (IS‐nPE) in human plasma

Compound	QC Level^a^	IS‐nME	IS‐nER	IS‐nPE
		% (RSD)	% (RSD)	% (RSD)
Bictegravir/[^13^C,^2^H_2_,^15^N]‐Bictegravir	Low	0 (6)	−6 (6)	−6 (7)
	Middle	3 (3)	−5 (3)	−2 (2)
	High	−5 (3)	−8 (4)	−12 (3)
Cabotegravir/[^13^C, ^2^H_5_]‐Cabotegravir	Low	5 (4)	−6 (6)	−1 (5)
	Middle	−1 (3)	−6 (4)	−6 (3)
	High	−6 (3)	−7 (3)	−12 (3)
Doravirine/[^13^C_6_]‐Doravirine	Low	3 (5)	−11 (5)	−8 (3)
	Middle	4 (3)	−8 (3)	−5 (2)
	High	−4 (2)	−10 (3)	−13 (3)
Rilpivirine/[^13^C_6_]‐Rilpivirine	Low	12 (4)	−15 (10)	−5 (8)
	Middle	9 (5)	−4 (8)	5 (3)
	High	−6 (3)	−11 (6)	−16 (4)

Abbreviation: RSD, relative standard deviation.

a Low concentrations are defined as 60 ng/mL (12 ng/mL for rilpivirine), middle concentrations are defined as 600 ng/mL (120 ng/mL for rilpivirine), and high concentrations are defined as 6000 ng/mL (1200 ng/mL for rilpivirine).

The lack of significant ME was corroborated by the standard line slopes approach, with RSD values of 2.0%, 1.7%, 2.3%, and 1.8% for bictegravir, cabotegravir, doravirine, and rilpivirine, respectively.

#### 3.2.3 Trueness, precision, and accuracy profile

Analyte/IS peak area ratios were plotted vs analyte concentrations to obtain response functions. The quadratic log‐log regression model provided the best description of the response‐concentration profile in terms of determination coefficient and back‐calculated calibration samples (±15% and ±20% at expected LLOQ) and was finally retained for each compound. For each series, plasma levels of the validation standards were then calculated using the calibration curves. The validated calibration ranges varied from 25 to 10 000 ng/mL for bictegravir, cabotegravir, and doravirine and from 10 to 2000 ng/mL for rilpivirine. Trueness (94.7% to 107.5%), repeatability (2.6% to 11.0%), and intermediate precision (3.0% to 11.2%) were appropriate for quantifying plasma levels of the four ARV drugs of interest (Table 3).

**Table 3:** Trueness, repeatability, and intermediate precision in human plasma over the validated range

Compound	Concentration, ng/mL	Trueness, %	Precision		Relative Uncertainty, %
			Repeatability, %	Intermediate Precision, %	
Bictegravir	30	106.2	9.6	9.6	27.2
	60	107.5	6.2	6.2	17.6
	150	103.1	7.8	7.8	22.0
	300	97.8	3.4	3.8	11.3
	600	96.6	6.0	6.0	15.2
	1500	97.0	4.0	4.1	11.6
	4500	96.5	4.3	4.3	12.1
	9000	101.6	6.5	6.5	18.4
Cabotegravir	30	94.7	11.0	11.2	41.4
	60	101.3	6.7	11.1	41.4
	150	97.7	5.6	8.5	29.8
	300	101.5	4.0	6.6	24.1
	600	99.8	2.6	3.0	9.0
	1500	103.9	4.3	4.3	12.2
	4500	97.1	3.5	3.5	10.0
	9000	97.7	3.5	3.5	9.9
Doravirine	30	104.0	4.0	4.0	11.2
	60	102.2	2.8	3.7	12.3
	150	100.8	3.4	3.4	9.5
	300	100.7	3.2	3.5	10.3
	600	100.2	3.0	4.3	14.8
	1500	102.2	3.6	4.4	14.0
	4500	97.9	3.3	3.3	9.3
	9000	99.4	3.1	3.3	9.6
Rilpivirine	12	100.9	7.6	7.6	21.4
	30	107.5	5.4	6.2	18.8
	60	100.7	6.9	6.9	19.4
	120	101.5	4.3	4.3	12.1
	300	106.3	4.5	4.8	14.0
	900	101.1	3.7	3.9	11.3
	1800	100.1	3.7	3.9	11.1

**Figure 4:**
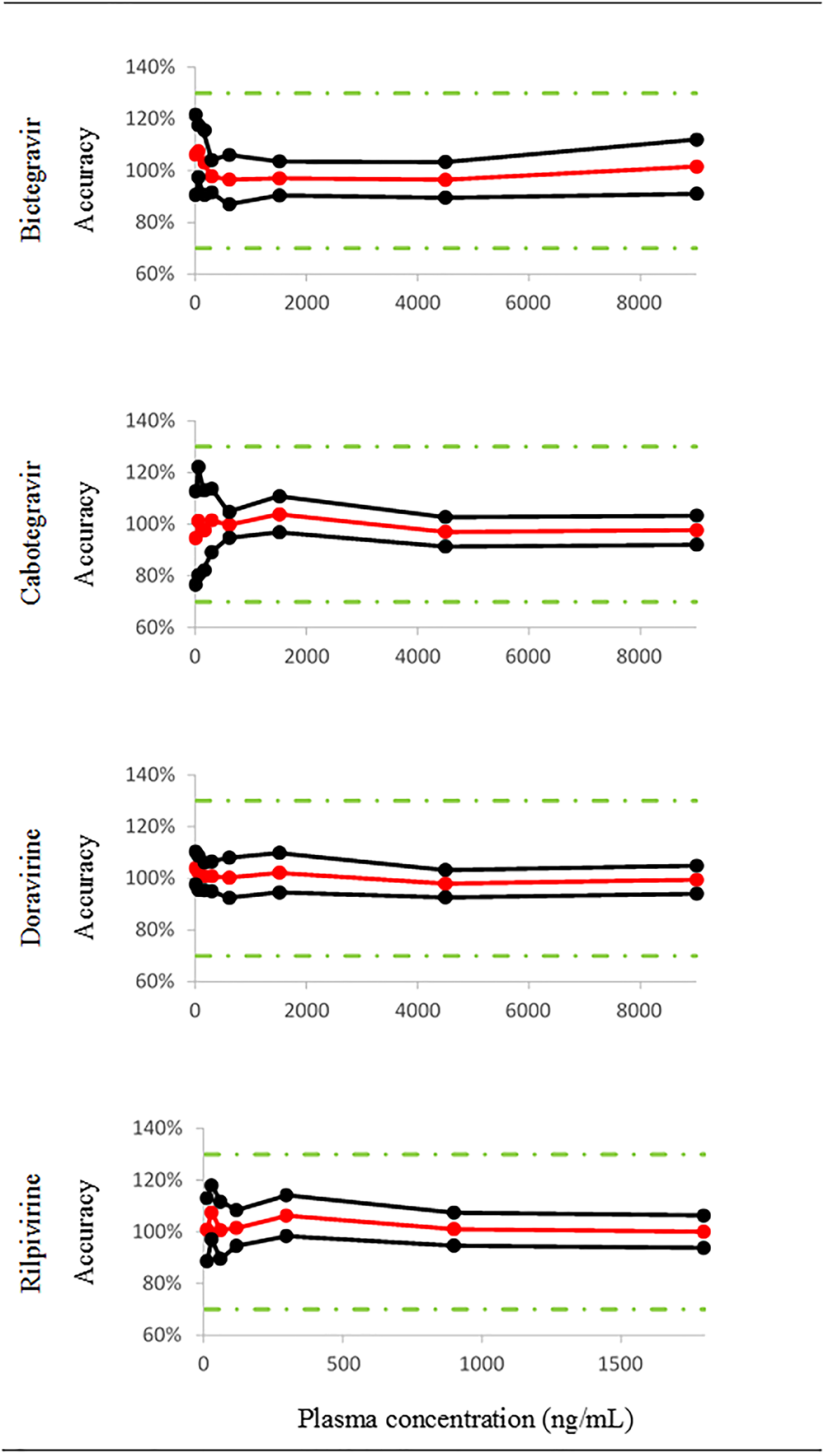
Accuracy profiles over the validated domain in human plasma of the five comedications and the two metabolites. Trueness (red solid line), upper and lower β‐expectation tolerance intervals (*β* = 80%) (black solid lines) and acceptance limits (λ = ±30%, green dotted lines) are shown [Colour figure can be viewed at wileyonlinelibrary.com]

A *β* value of 80% was chosen for the establishment of β‐expectation tolerance intervals, representing the fraction of future results that would be expected to fall within the obtained tolerance intervals during routine application of the method.^46^ As demonstrated in Figure 4, accuracy profiles obtained for each compound lie within the acceptance limits of ±30% for biological samples.^26^

Since the β‐expectation tolerance interval of bictegravir, cabotegravir, and doravirine does not cross the acceptance limits of ±30%, the LLOQ was defined as the concentration of the lowest validation sample (ie, 30 ng/mL). Considering an accuracy of ±30% (total error), the lowest concentration measurable in human plasma (LLOQ) was 10 ng/mL for rilpivirine.

Linearity was considered satisfactory since slopes and intercepts ranged from 0.96 to 1.03 and −75.4 to 40.2, respectively. In addition, determination coefficient (*R* ^2^) were all higher than .99.

#### 3.2.4 Measurement uncertainty

The absolute uncertainty vs concentration profiles were best described by polynomial (bictegravir, cabotegravir, and doravirine) and power regression models (rilpivirine).

The relative uncertainty of each compound at each validation levels is shown in Table 3. With a confidence level of 95%, the unknown true value located at maximum ±27.2%, ±41.4%, ±14.8%, and ±21.4% around the measured result for bictegravir, cabotegravir, doravirine, and rilpivirine, respectively. Table 3 also demonstrates that the relative uncertainty is higher at the lowest concentrations of the validation domain.

#### 3.2.5 Stability studies

Stability of analytes in plasma is reported in Table 4. Results demonstrated that analytes did not significantly degrade after storage of plasma samples at room temperature and +4°C for up to 48 hours. In addition, no significant alteration of plasma concentrations was observed after three consecutive freeze‐thaw cycles (variation <±15%). The thermal viro‐inactivation process had no significant influence on analytes concentrations, with variations comprised between −6.5% and 10.6%. Finally, medium‐term stability studies showed no significant influence of degradation after 6 weeks of freezing at −20°C and −80°C.

**Table 4:** Stability studies

Compound	QC Concentration (ng/mL)	Room Temperature for 24 h	Room Temperature for 48 h	+4°C for 24 h	+4°C for 48 h	After 3 Consecutive Freeze‐thaw Cycles	Thermal Viro‐inactivation^a^	−2°C During 6 weeks	−80°C During 6 weeks
Bictegravir	60	−8.0	−10.4	−4.8	−9.3	−10.5	−6.3	−8.2	−11
	600	6.7	11.4	3.8	−4.3	−0.9	−2.6	8.3	11.9
	6000	5.1	9.6	4.9	0.7	−5.9	10.4	10.0	1.0
Cabotegravir	60	−5.4	−9.5	−10.3	−5.9	−12.9	−6.4	3.1	12.9
	600	2.8	5.0	10.1	5.0	8.2	9.8	14.4	13.6
	6000	6.2	6.8	6.3	7.3	6.4	7.1	12.5	13.4
Doravirine	60	−7.0	−6.2	−2.6	−6.4	−7.6	3.2	−8.9	−5.0
	600	−2.1	0.0	1.9	3.7	2.2	7.1	8.0	9.6
	6000	−3.0	3.9	4.0	0.0	0.5	2.7	−0.7	1.9
Rilpivirine	12	8.9	14.5	12.9	11.9	−2.1	10.5	−11.4	−5.3
	120	11.3	9.4	8.8	10.7	2.9	7.2	10.8	11.9
	1200	2.4	8.8	7.9	6.4	−0.3	1.9	5.4	2.1

*Note.* Data are reported as deviations (%) from concentration measured at *t*_0_.

a 60 min at 60°C in a water bath.

### 3.3 Clinical applications

**Figure 5:**
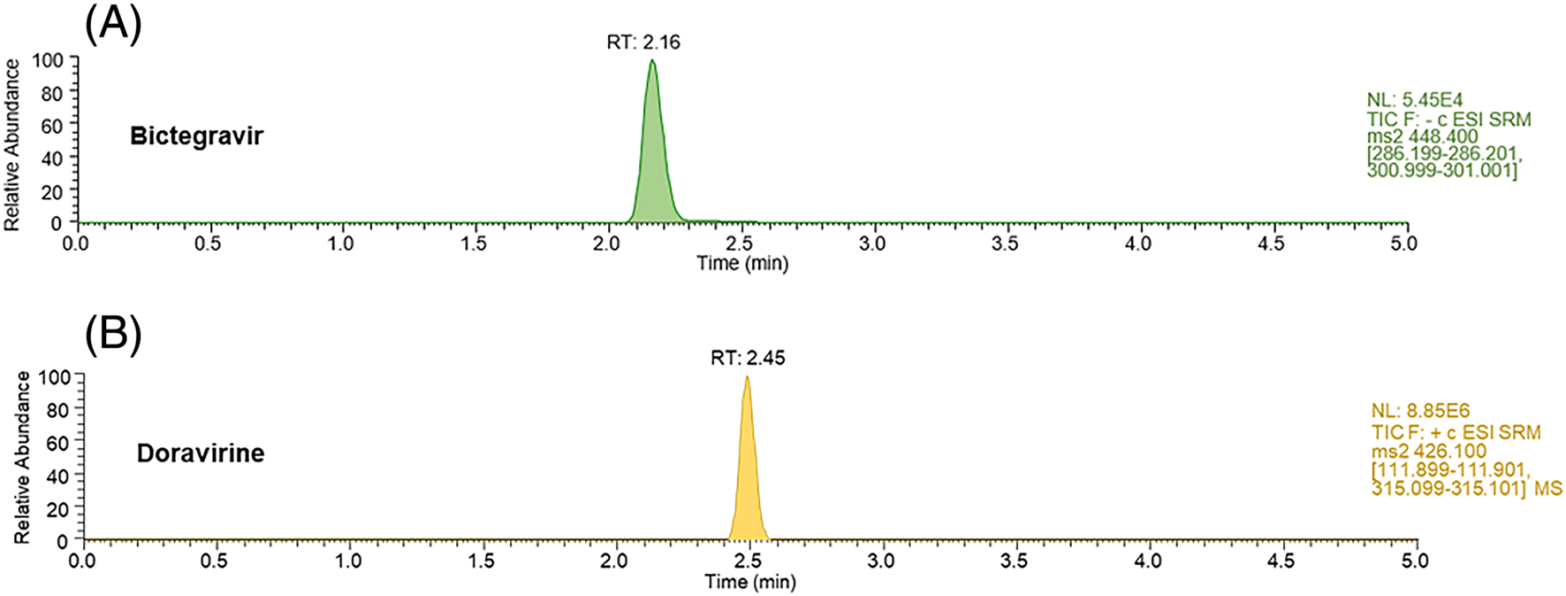
Chromatographic profile of a plasma from one HIV‐infected individual receiving bictegravir 50 mg once daily (A) and from another patient receiving doravirine 100 mg once daily (B) [Colour figure can be viewed at wileyonlinelibrary.com]

The proposed LC‐MS/MS assay has been applied to patient's samples obtained for clinical purposes, within the framework of our TDM service. A typical chromatographic profile of a plasma from an HIV‐infected patient receiving bictegravir 50 mg once daily is shown in Figure 5A. Plasma sample was collected 17.25 hours after the last drug intake, and plasma concentration of bictegravir was 3351 ± 340 ng/mL.

**Figure 6:**
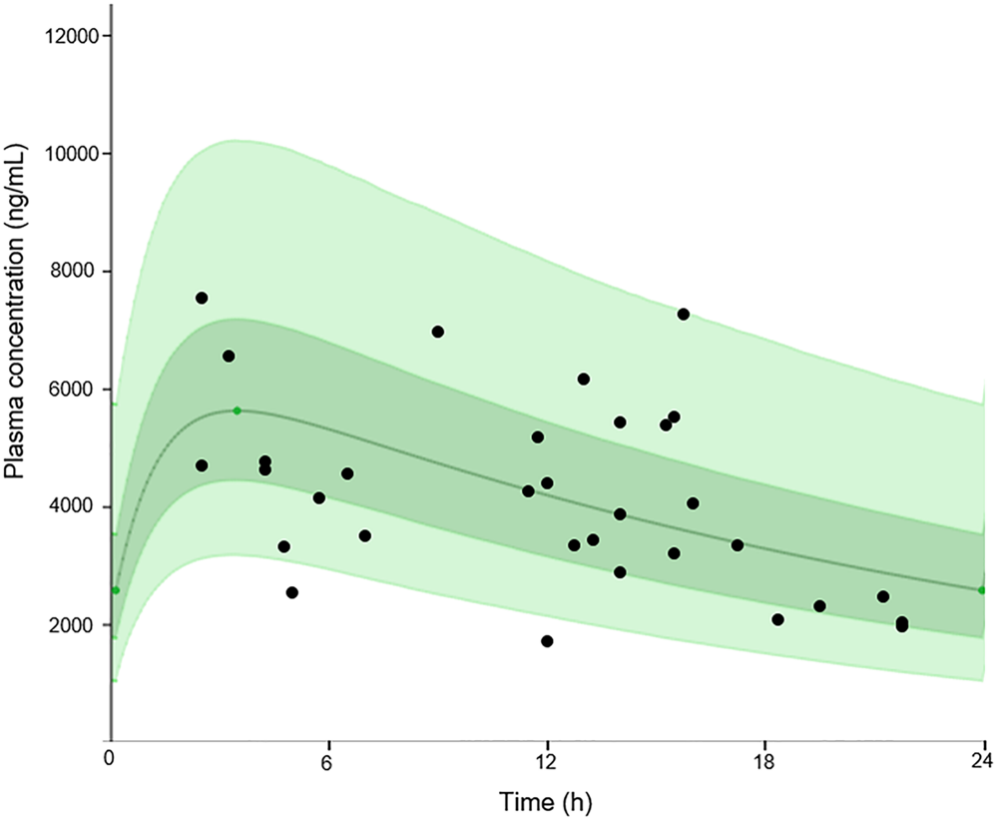
Steady‐state pharmacokinetic profile of bictegravir when administered at 50 mg once daily. Continuous green line represents the population median prediction. Dark green and light green shaded area represent the 50% and 90% prediction interval, respectively. Bictegravir plasma concentrations obtained from our therapeutic drug monitoring (TDM) service in patients included in the Swiss HIV Cohort Study has been superimposed (black points) [Colour figure can be viewed at wileyonlinelibrary.com]

At present, 32 plasma samples from PLWH receiving bictegravir have been collected in our TDM service. The interpretation of these plasma concentrations was made possible thanks to the availability of a population pharmacokinetic model summarized in the European Public Assessment Report.^47^, ^48^ Using the Tucuxi software^49^, ^50^ developed by our service, the pharmacokinetic profile of bictegravir at steady state was simulated over the dosing interval, exploiting the reported intraindividual and interindividual variabilities. The 32 plasma concentrations determined using the proposed LC‐MS/MS assay were compared with the simulated population pharmacokinetic profile to ascertain the expectedness of the result. As shown in Figure 6, 59% (n = 19) and 94% (n = 30) of the measured bictegravir plasma concentrations lied into the 50% and 95% prediction interval, respectively. This result demonstrates the ability of our LC‐MS/MS methodology to quantify bictegravir and to replicate the manufacturer's findings regarding the rather large variability of plasma concentrations commonly observed in clinical practice.

In addition, the chromatographic profile of an HIV‐infected individual receiving doravirine 100 mg once daily is shown in Figure 5B. Doravirine plasma concentration measured in this patient was 1139 ± 124 ng/mL, 15 hours after the last drug intake.

Finally, the combination of LAI cabotegravir/rilpivirine, injected monthly, has been demonstrated as effective as the daily, oral, three‐drug regimen in maintaining HIV virus suppression throughout a 48‐week period.^9^, ^10^, ^51^ The LAI cabotegravir/rilpivirine formulation (Cabenuva) is therefore currently being reviewed by the Food and Drug Administration. Once used in the clinical setting, it is anticipated that physicians will be asking for cabotegravir and rilpivirine levels measurement in patients on LAI cabotegravir/rilpivirine, for the monitoring of their plasma drug exposure in special clinical situations such as the initiation of treatments for inaugural diseases with definite risk of DDIs (tuberculosis, epilepsy, HCV infection, or cancer). Finally, although no clear correlation has been established between cabotegravir and rilpivirine plasma concentrations and the emergence of resistance during the phase 2 study LATTE‐2,^14^ the management of failure of ARV drug remains crucial.^52^ This bioanalytical assay offers clinicians the possibility to closely monitor the plasma levels of cabotegravir and rilpivirine in the special instances where LAI‐ARV drug needs to be stopped and switched to oral intake of ARV drugs.

## 4 CONCLUSION

A sensitive and selective LC‐MS/MS assay was developed and validated, enabling the simultaneous quantification in human plasma of four newly approved ARV agents, or ARV drugs at the latest phase of their development. Validation performances met international recommendations for bioanalytical assay and were achieved over a large validation domain that covers the plasma concentrations commonly observed in clinical practice. The method could be easily implemented for both clinical and research purposes. Our assay thus provides important information on the plasma levels of these latest generation ARV drugs in PLWH patients and constitutes a useful TDM tool for ascertaining that they are always exposed to suitable systemic drug exposure in the various clinical situations that do occur in the real‐life conditions.

## CONFLICT OF INTEREST

No conflicts of interest to declare. M.C., outside of this study, has received through his institution research grant from ViiV, Gilead, and offered expert testimony for Abbvie, MSD, Gilead, and Sandoz. H.F.G., outside of this study, received unrestricted research grants from Gilead and Roche; fees for data and safety monitoring board membership from Merck; and consulting/advisory board membership fees from Merck, ViiV, and Gilead.
